# Bis[*N*-(3-amino­prop­yl)propane-1,3-di­amine-κ^3^ *N*,*N*′,*N*′′]cadmium nitrate perchlorate

**DOI:** 10.1107/S1600536812004400

**Published:** 2012-02-10

**Authors:** Hamid Goudarziafshar, Mohsen Nikoorazm, Yunes Abbasityula, Václav Eigner, Michal Dušek

**Affiliations:** a Faculty of Science, Department of Chemistry, Ilam University, Ilam, Iran; b Department of Solid State Chemistry, Institute of Chemical Technology, Technická 5, 166 28 Prague, Czech Republic; c Institute of Physics AS CR, v.v.i., Na Slovance 2, 182 21 Prague 8, Czech Republic

## Abstract

The title complex, [Cd(C_6_H_17_N_3_)_2_](ClO_4_)(NO_3_), was synthesized by the reaction of Cd(NO_3_)_2_·4H_2_O, bis­(3-amino­prop­yl)­amine and sodium perchlorate in methanol. The asymmetric unit of the title complex consists of one Cd^2+^ cation, two tridentate bis­(3-amino­prop­yl)amine ligands, one nitrate anion and one perchlorate anion. The Cd^2+^ cation is coordinated by six N atoms of the bis­(3-amino­prop­yl)amine ligands in a slightly distorted octa­hedral coordination geometry. In the crystal, mol­ecules are held together by an intricate network of N—H⋯O inter­actions. One of the two amine ligands was found to be disordered over two sets of sites, with a ratio of 0.802 (3):0.198 (3), similarly to the nitrate anion, with a ratio of 0.762 (10):0.238 (10).

## Related literature

For background about the usage of this ligand for complexation, see: Boeckmann & Näther (2011*a* ▶,*b* ▶); Choi *et al.* (1995 ▶); Pajunen *et al.* (1996 ▶); Maji *et al.* (2003 ▶). For the extinction correction, see: Becker & Coppens (1974 ▶).
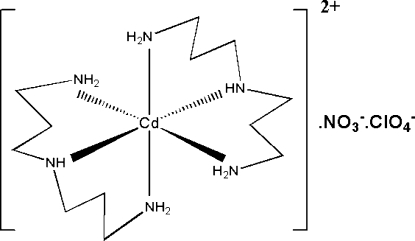


## Experimental

### 

#### Crystal data

[Cd(C_6_H_17_N_3_)_2_](NO_3_)(ClO_4_)
*M* *_r_* = 536.3
Monoclinic, 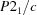

*a* = 12.6030 (5) Å
*b* = 11.9403 (5) Å
*c* = 14.1977 (5) Å
β = 97.717 (3)°
*V* = 2117.17 (14) Å^3^
*Z* = 4
Cu *K*α radiation
μ = 9.86 mm^−1^
*T* = 120 K
0.35 × 0.30 × 0.21 mm

#### Data collection

Oxford Diffraction CCD diffractometer
Absorption correction: analytical (Clark & Reid, 1995 ▶) *T*_min_ = 0.103, *T*_max_ = 0.286
53531 measured reflections
3778 independent reflections
3680 reflections with *I* > 3σ(*I*)
*R*_int_ = 0.038

#### Refinement

*R*[*F* ^2^ > 3σ(*F* ^2^)] = 0.022
*wR*(*F* ^2^) = 0.068
*S* = 1.50
3778 reflections
298 parameters
14 restraints
H atoms treated by a mixture of independent and constrained refinement
Δρ_max_ = 0.28 e Å^−3^
Δρ_min_ = −0.43 e Å^−3^

### 

Data collection: *CrysAlis PRO* (Agilent, 2011 ▶); cell refinement: *CrysAlis PRO*; data reduction: *CrysAlis PRO*; program(s) used to solve structure: *SUPERFLIP* (Palatinus & Chapuis, 2007 ▶); program(s) used to refine structure: *JANA2006* (Petříček *et al.*, 2006 ▶); molecular graphics: *ORTEP-3* (Farrugia, 1997 ▶) and *Mercury* (Macrae *et al.*, 2008 ▶); software used to prepare material for publication: *publCIF* (Westrip, 2010 ▶).

## Supplementary Material

Crystal structure: contains datablock(s) global, I. DOI: 10.1107/S1600536812004400/wm2586sup1.cif

Structure factors: contains datablock(s) I. DOI: 10.1107/S1600536812004400/wm2586Isup2.hkl

Additional supplementary materials:  crystallographic information; 3D view; checkCIF report

## Figures and Tables

**Table 1:** Selected bond lengths (Å)

Cd1—N1	2.3456 (16)
Cd1—N2	2.5084 (14)
Cd1—N3	2.3430 (19)
Cd1—N4	2.3598 (17)
Cd1—N5	2.3970 (17)
Cd1—N7	2.426 (2)

**Table 2:** Hydrogen-bond geometry (Å, °)

*D*—H⋯*A*	*D*—H	H⋯*A*	*D*⋯*A*	*D*—H⋯*A*
N1—H1n1⋯O5*a*^i^	0.870 (17)	2.289 (19)	3.127 (6)	161.7 (16)
N1—H1n1⋯O5*b*^i^	0.870 (17)	2.10 (2)	2.929 (10)	158.1 (16)
N1—H2n1⋯O7*a*^ii^	0.870 (12)	2.278 (17)	2.986 (7)	139 (2)
N1—H2n1⋯O4*b*^ii^	0.870 (12)	2.403 (14)	3.262 (9)	170 (2)
N1—H2n1⋯O7*b*^ii^	0.870 (12)	2.410 (19)	3.027 (11)	128.4 (19)
N2—H1n2⋯O4*a*^i^	0.870 (17)	2.339 (18)	3.149 (4)	155.0 (18)
N2—H1n2⋯O4*b*^i^	0.870 (17)	2.28 (2)	3.116 (10)	162.4 (16)
N3—H1n3⋯O3^iii^	0.870 (19)	2.43 (2)	3.196 (3)	148 (2)
N3—H2n3⋯O5*a*^iv^	0.870 (9)	2.472 (13)	3.289 (8)	157 (2)
N3—H2n3⋯O7*a*^iv^	0.870 (9)	2.48 (2)	3.134 (7)	132 (2)
N3—H2n3⋯O5*b*^iv^	0.870 (9)	2.426 (18)	3.215 (13)	151 (2)
N3—H2n3⋯O7*b*^iv^	0.870 (9)	2.50 (2)	3.239 (13)	144 (2)
N5—H1n5⋯O4*a*^i^	0.870 (17)	2.396 (16)	3.200 (4)	154 (2)
N5—H1n5⋯O5*a*^i^	0.870 (17)	2.30 (2)	3.065 (7)	146.5 (16)
N5—H1n5⋯O5*b*^i^	0.870 (17)	2.33 (2)	3.076 (12)	144.0 (16)
N5—H2n5⋯O1^v^	0.870 (14)	2.263 (14)	3.122 (2)	169 (2)
N7—H1n7⋯O4*a*^ii^	0.87	2.20	3.067 (4)	174.11
N7—H1n7⋯O4*b*^ii^	0.87	2.45	3.313 (11)	169.55
N4—H1n4⋯O3^iii^	0.870 (5)	2.379 (11)	3.215 (2)	161 (2)
N4—H2n4⋯O2^v^	0.870 (15)	2.170 (15)	3.011 (2)	163 (2)

Symmetry codes: (i) 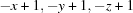; (ii) 
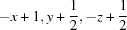
; (iii) 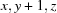; (iv) 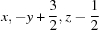; (v) 
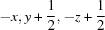
.

**Fig. 1.**
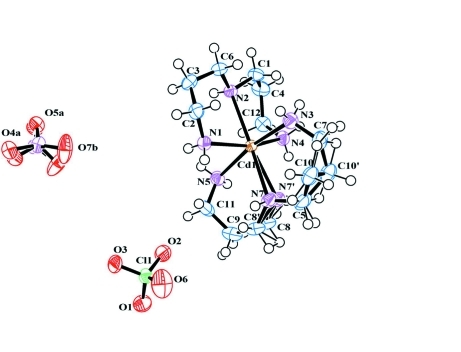
The title compound with displacement ellipsoids drawn at the 50% probability level. H atoms are shown as spheres of arbitary radius.

**Fig. 2.**
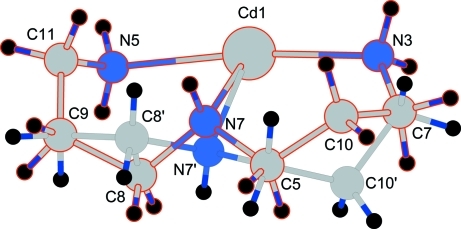
Disorder of dpt. The chain of dpt assumes two conformations with occupations refined to 0.802 (3) and 0.198 (3), respectively. The atoms and bonds of the major part are highlighted in red. Some atoms of both conformers coincide.

**Fig. 3.**
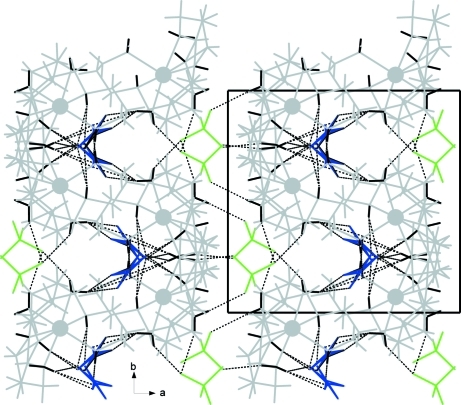
Packing of the title structure with hydrogen bonds. Molecules of the complex are plotted in light gray, cadmium is a gray circle. Perchlorate anion and nitrate anion are highlighted in light green and blue, respectively. N—H bonds in black thick lines, N—H···O hydrogen bonds in black dashed lines.

## supplementary crystallographic information

### Comment

In the crystal structure of the title complex, see Fig. 1, the Cd^2+^ cation is bonded to six nitrogen atoms of two tridentate bis(3-aminopropyl)amine ligands in a slightly distorted octahedral coordination. One of the ligands was found to be disordered, assuming two differently occupied orientations (Fig. 2). Because the low occupation of one of these orientations does not allow its free refinement, its precise geometry remains unsure (for further details, see experimental section) and therefore will not be included in the following discussion.

The octahedral coordination sphere around Cd^2+^ contains two longer Cd–N2 and Cd–N7 bonds of 2.5084 (14) and 2.426 (2) Å as well as two shorter Cd–N3 and Cd–N1 bonds of 2.3430 (19) and 2.3456 (16) Å, respectively (Table 1). The angles around the metals atoms range from 81.40 (5)° to 103.19 (7)° and from 159.83 (5)° to 175.31 (7)°. Both the perchlorate and nitrate anions are linked to the complex cations through an intricate network of N—H···O hydrogen bonds. The hydrogen bonds involving the nitrate anion connect the molecules of the complex to slabs parallel to (100). The hydrogen bonds involving the perchlorate anion eventually connect the slabs into three-dimensional network (Table 2, Fig. 3).

Similar complexes with bis(3-aminopropyl)amine as a ligand were reported previously, e.g. by Boeckmann & Näther (2011**a*,b*); Choi *et al.* (1995); Maji *et al.*(2003); Pajunen *et al.* (1996).

### Experimental

The title complex was prepared by the branch tube method: bis(3-aminopropyl)amine (0.282 ml, 2 mmol) was placed in one arm of a branched tube and Cd(NO_3_)_2_.4H_2_O (0.308 g, 1 mmol) and sodium perchlorate (0.122 g, 1 mmol) in the other. Methanol was then carefully added to fill both arms, the tube sealed and the ligand-containing arm immersed in a bath at 333 K, while the other was left at ambient temperature. After one week, colorless crystals were collected in the cooler arm. Then they were then filtered off, washed with acetone and diethylether, and air dried. M.p.: 583 K, yield: (78%).

### Refinement

All hydrogen atoms were discernible in difference Fourier maps and could be refined to reasonable geometries. According to common practice, H atoms bonded to C were kept in ideal positions with C–H = 0.96 Å while positions of H atoms bonded to N (except N7 and N7' – see below) were refined with distance restraint N–H= 0.87 Å (NH_1_, NH_2_). In both cases *U*_iso_(H) was set to 1.2*U*_eq_(C,*N*). Disorder of the nitrate anion was treated using a rigid body refinement. Two orientations of the disordered bis(3-aminopropyl)amine ligand (Fig. 2) were refined using split atom positions. While some atoms of both orientations coincide, the C8, N7 and C10 positions were split to C8—C8', N7—N7' and C10—C10', respectively. The sum of occupancies for each split pair was kept equal to the original full occupancy and the occupancies of all atoms belonging to a given conformer were kept equal. Positions C5, C7 and C9 were the same for both orientations but the attached hydrogen atoms positions were split because of a different geometry of neighboring atoms. These carbon atoms were formally split by placing two atoms to the same position in order to allow the refinement program using two geometry constraints for two different pairs of hydrogen atoms. Thus C5 is bonded to hydrogen atoms H1C5, H2C5, H1C5' and H2C5', and similarly for C7 and C9. The positions of the major orientation were refined without restrictions. However, the low occupancy of the minor component, 0.198 (3), did not allow a free refinement of positions C8', N7', and C10'. For these atoms we defined following distance restraints: C9–C8' = 1.517 Å, C8'–N7' = 1.482 Å, N7'–C5' = 1.482 Å, C5'–C10' = 1.517 Å and C10'–C7 = 1.517 Å, with weight 0.001 allowing only very small deviations during the refinement from the defined values. Hydrogen atoms attached to N7 and N7' were kept in ideal positions, without refinement.

The angles of the minor component of the disordered part were refined to slightly differents values compared with the major part (see Table 2). However, it remained unclear whether the difference could be taken seriously or whether this is caused by an unreliable refinement due to the low occupation. For this reason we created (using the rigid body tool in Jana2006) a structure model where both orientations were described with a common shape of the ligand refining its atomic parameters and a translation vector plus three rotations (followed by inversion, when necessary) allowing transformation of the common ligand to the position of the first and the second orientation, respectively. In this approach, both orientations have exactly the same geometry, differing only in their occupation. While the number of refined parameters decreased from 298 to 295, the *R* value increased by 0.8% and the occupation of the minor component decreased from 0.198 (3) to 0.109 (4). The increase of *R* value and decrease of the minor component occupation indicate that the conformers are really slightly different in their shape. Hence this approach was finally neglected.

### Crystal data

**Table:**

[Cd(C_6_H_17_N_3_)_2_](NO_3_)(ClO_4_)	*F*(000) = 1104
*M**_r_* = 536.3	*D*_x_ = 1.682 Mg m^−^^3^
Monoclinic, *P*2_1_/*c*	Melting point: 210 K
Hall symbol: -P 2ycb	Cu *K*α radiation, λ = 1.5418 Å
*a* = 12.6030 (5) Å	Cell parameters from 35544 reflections
*b* = 11.9403 (5) Å	θ = 3.1–67.0°
*c* = 14.1977 (5) Å	µ = 9.86 mm^−^^1^
β = 97.717 (3)°	*T* = 120 K
*V* = 2117.17 (14) Å^3^	Parallelpiped, colourless
*Z* = 4	0.35 × 0.30 × 0.21 mm

### Data collection

**Table:**

Oxford Diffraction CCD diffractometer	3778 independent reflections
Radiation source: X-ray tube	3680 reflections with *I* > 3σ(*I*)
Graphite monochromator	*R*_int_ = 0.038
Detector resolution: 10.3784 pixels mm^-1^	θ_max_ = 67.1°, θ_min_ = 3.5°
ω scans	*h* = −15→15
Absorption correction: analytical (Clark & Reid, 1995)	*k* = −14→14
*T*_min_ = 0.103, *T*_max_ = 0.286	*l* = −16→16
53531 measured reflections	

### Refinement

**Table:**

Refinement on *F*^2^	Hydrogen site location: inferred from neighbouring sites
*R*[*F*^2^ > 2σ(*F*^2^)] = 0.022	H atoms treated by a mixture of independent and constrained refinement
*wR*(*F*^2^) = 0.068	Weighting scheme based on measured s.u.'s *w* = 1/(σ^2^(*I*) + 0.0016*I*^2^)
*S* = 1.50	(Δ/σ)_max_ = 0.039
3778 reflections	Δρ_max_ = 0.28 e Å^−^^3^
298 parameters	Δρ_min_ = −0.43 e Å^−^^3^
14 restraints	Extinction correction: B-C type 1 Gaussian isotropic (Becker & Coppens, 1974)
232 constraints	Extinction coefficient: 2810 (150)

### Fractional atomic coordinates and isotropic or equivalent isotropic displacement parameters (Å^2^)

**Table:**

	*x*	*y*	*z*	*U*_iso_*/*U*_eq_	Occ. (<1)
Cd1	0.277764 (9)	0.929182 (10)	0.204521 (8)	0.01647 (7)	
Cl1	0.10390 (3)	0.32823 (4)	0.20236 (3)	0.02461 (14)	
O1	0.02248 (12)	0.24458 (15)	0.18695 (12)	0.0392 (5)	
O2	0.07450 (15)	0.41160 (16)	0.26595 (13)	0.0443 (6)	
N1	0.43298 (12)	0.82683 (14)	0.18776 (11)	0.0207 (4)	
N2	0.38913 (12)	0.98590 (13)	0.35687 (11)	0.0193 (4)	
N3	0.32575 (14)	1.09505 (16)	0.13282 (12)	0.0251 (5)	
O3	0.20171 (12)	0.27577 (14)	0.24324 (13)	0.0402 (5)	
N4	0.14304 (12)	1.01825 (14)	0.27775 (12)	0.0229 (5)	
N5	0.20751 (13)	0.75584 (14)	0.25474 (12)	0.0241 (5)	
C1	0.34052 (17)	1.08536 (17)	0.39575 (15)	0.0253 (6)	
O6	0.11755 (18)	0.3793 (2)	0.11419 (15)	0.0598 (8)	
N7	0.18359 (18)	0.87035 (19)	0.05226 (15)	0.0232 (6)	0.802 (3)
C2	0.53983 (15)	0.87914 (18)	0.21111 (14)	0.0240 (6)	
C3	0.56460 (16)	0.91132 (18)	0.31510 (15)	0.0260 (6)	
C4	0.23878 (19)	1.06025 (18)	0.43935 (16)	0.0304 (7)	
C5	0.15261 (18)	0.9619 (2)	−0.01547 (15)	0.0333 (7)	1.000 (3)
C6	0.50246 (14)	1.01040 (17)	0.34683 (13)	0.0232 (5)	
C7	0.26605 (18)	1.1318 (2)	0.04151 (15)	0.0344 (7)	1.000 (3)
C8	0.0878 (2)	0.8049 (2)	0.06677 (17)	0.0288 (8)	0.802 (3)
C9	0.11010 (18)	0.68513 (19)	0.10329 (16)	0.0346 (7)	1.000 (3)
C10	0.2447 (2)	1.0412 (3)	−0.03105 (19)	0.0317 (8)	0.802 (3)
C11	0.20439 (18)	0.66885 (18)	0.17965 (17)	0.0358 (7)	
C12	0.15661 (15)	0.98729 (18)	0.37967 (14)	0.0261 (6)	
C8'	0.1370 (8)	0.7727 (5)	0.0329 (4)	0.0288 (8)	0.198 (3)
N7'	0.1439 (8)	0.8897 (5)	0.0681 (3)	0.0232 (6)	0.198 (3)
C10'	0.1547 (2)	1.0843 (3)	0.0133 (7)	0.0317 (8)	0.198 (3)
H1c1	0.32537	1.140614	0.346667	0.0304*	
H2c1	0.392061	1.120513	0.442423	0.0304*	
H1c2	0.544128	0.944375	0.172279	0.0289*	
H2c2	0.593786	0.82863	0.194848	0.0289*	
H1c3	0.554441	0.847495	0.354031	0.0312*	
H2c3	0.639995	0.925011	0.330538	0.0312*	
H1c4	0.257482	1.026754	0.500806	0.0365*	
H2c4	0.205839	1.129304	0.454383	0.0365*	
H1c6	0.538386	1.038912	0.405874	0.0279*	
H2c6	0.505454	1.071432	0.303248	0.0279*	
H1c11	0.200311	0.596067	0.207665	0.0429*	
H2c11	0.269576	0.671419	0.151642	0.0429*	
H1c12	0.177775	0.910171	0.386587	0.0313*	
H2c12	0.088988	0.993087	0.403398	0.0313*	
H1n1	0.4272 (18)	0.7680 (12)	0.2228 (14)	0.0248*	
H2n1	0.4284 (19)	0.8001 (19)	0.1304 (7)	0.0248*	
H1n2	0.393 (2)	0.9325 (14)	0.3989 (13)	0.0232*	
H1n3	0.319 (2)	1.1494 (14)	0.1725 (14)	0.0301*	
H2n3	0.3936 (5)	1.092 (2)	0.1270 (19)	0.0301*	
H1n5	0.2532 (15)	0.735 (2)	0.3028 (11)	0.0289*	
H2n5	0.1433 (8)	0.762 (2)	0.2701 (17)	0.0289*	
H1n7	0.229198	0.82966	0.026483	0.0278*	0.802 (3)
H1n7'	0.085949	0.904103	0.09297	0.0278*	0.198 (3)
H1n4	0.144 (2)	1.0907 (3)	0.2717 (19)	0.0275*	
H2n4	0.0813 (10)	0.992 (2)	0.2525 (16)	0.0275*	
H1c5	0.095396	1.004302	0.005448	0.0399*	0.802 (3)
H2c5	0.121722	0.931059	−0.075357	0.0399*	0.802 (3)
H1c8	0.048027	0.844456	0.10935	0.0346*	0.802 (3)
H2c8	0.039193	0.802801	0.008623	0.0346*	0.802 (3)
H1c10	0.308815	0.998445	−0.033304	0.0381*	0.802 (3)
H2c10	0.230607	1.073881	−0.093236	0.0381*	0.802 (3)
H1c8'	0.086071	0.768553	−0.023731	0.0346*	0.198 (3)
H2c8'	0.202668	0.752831	0.009863	0.0346*	0.198 (3)
H1c10'	0.11768	1.128123	−0.037477	0.0381*	0.198 (3)
H2c10'	0.113114	1.094335	0.064625	0.0381*	0.198 (3)
H1c5'	0.092521	0.948654	−0.063287	0.0399*	0.198 (3)
H2c5'	0.217125	0.94398	−0.041301	0.0399*	0.198 (3)
H1c9	0.046796	0.655002	0.124538	0.0415*	0.802 (3)
H2c9	0.117273	0.636468	0.050693	0.0415*	0.802 (3)
H1c7	0.304021	1.191874	0.015936	0.0413*	0.802 (3)
H2c7	0.199552	1.165233	0.05257	0.0413*	0.802 (3)
H1c7'	0.308636	1.118778	−0.008627	0.0413*	0.198 (3)
H2c7'	0.262893	1.21214	0.03986	0.0413*	0.198 (3)
H1c9'	0.049114	0.709287	0.131819	0.0415*	0.198 (3)
H2c9'	0.09353	0.615533	0.070763	0.0415*	0.198 (3)
O4a	0.6475 (3)	0.2430 (2)	0.5421 (3)	0.0291 (8)	0.762 (10)
O5a	0.5820 (6)	0.3444 (5)	0.6479 (4)	0.0268 (9)	0.762 (10)
N6a	0.5753 (4)	0.3067 (4)	0.5645 (4)	0.0251 (6)	0.762 (10)
O7a	0.4980 (5)	0.3311 (7)	0.5053 (4)	0.0549 (13)	0.762 (10)
O4b	0.6175 (9)	0.2377 (9)	0.5309 (6)	0.0291 (8)	0.238 (10)
O5b	0.5721 (10)	0.3276 (10)	0.6526 (7)	0.0268 (9)	0.238 (10)
N6b	0.5711 (10)	0.3192 (9)	0.5641 (6)	0.0251 (6)	0.238 (10)
O7b	0.5243 (10)	0.3897 (11)	0.5103 (7)	0.0549 (13)	0.238 (10)

### Atomic displacement parameters (Å^2^)

**Table:**

	*U*^11^	*U*^22^	*U*^33^	*U*^12^	*U*^13^	*U*^23^
Cd1	0.01634 (11)	0.01748 (12)	0.01549 (11)	−0.00038 (4)	0.00177 (6)	−0.00021 (4)
Cl1	0.0209 (2)	0.0243 (2)	0.0291 (2)	0.00155 (16)	0.00491 (17)	0.00203 (17)
O1	0.0245 (7)	0.0393 (9)	0.0532 (9)	−0.0073 (7)	0.0026 (6)	−0.0014 (7)
O2	0.0418 (10)	0.0455 (10)	0.0421 (10)	0.0168 (8)	−0.0069 (8)	−0.0160 (8)
N1	0.0221 (7)	0.0211 (8)	0.0190 (7)	0.0014 (6)	0.0038 (6)	−0.0003 (6)
N2	0.0201 (7)	0.0201 (8)	0.0175 (7)	−0.0022 (6)	0.0016 (6)	−0.0002 (6)
N3	0.0259 (8)	0.0252 (8)	0.0242 (8)	−0.0017 (7)	0.0035 (7)	0.0045 (7)
O3	0.0219 (7)	0.0335 (9)	0.0625 (11)	0.0065 (6)	−0.0042 (7)	−0.0024 (7)
N4	0.0210 (8)	0.0213 (9)	0.0267 (8)	0.0011 (6)	0.0044 (6)	0.0017 (6)
N5	0.0228 (8)	0.0212 (8)	0.0282 (9)	−0.0014 (7)	0.0033 (6)	0.0021 (7)
C1	0.0254 (10)	0.0253 (10)	0.0254 (10)	−0.0025 (8)	0.0039 (8)	−0.0072 (8)
O6	0.0699 (13)	0.0626 (14)	0.0530 (12)	0.0087 (11)	0.0303 (10)	0.0269 (10)
N7	0.0203 (12)	0.0291 (11)	0.0196 (10)	0.0044 (9)	0.0009 (8)	−0.0033 (8)
C2	0.0190 (9)	0.0269 (11)	0.0271 (10)	0.0024 (7)	0.0065 (7)	−0.0008 (8)
C3	0.0176 (9)	0.0322 (11)	0.0275 (10)	−0.0008 (8)	0.0003 (8)	0.0012 (8)
C4	0.0353 (12)	0.0335 (12)	0.0244 (11)	−0.0012 (9)	0.0111 (9)	−0.0093 (8)
C5	0.0326 (11)	0.0419 (12)	0.0222 (10)	0.0013 (10)	−0.0080 (8)	0.0036 (9)
C6	0.0202 (9)	0.0266 (10)	0.0224 (9)	−0.0049 (7)	0.0007 (7)	−0.0028 (7)
C7	0.0367 (11)	0.0316 (12)	0.0332 (11)	0.0004 (9)	−0.0017 (9)	0.0122 (9)
C8	0.0246 (12)	0.0373 (15)	0.0238 (12)	−0.0072 (11)	0.0004 (9)	−0.0080 (10)
C9	0.0346 (11)	0.0315 (12)	0.0380 (12)	−0.0100 (9)	0.0062 (9)	−0.0125 (9)
C10	0.0343 (14)	0.0395 (15)	0.0208 (12)	0.0030 (12)	0.0012 (10)	0.0070 (11)
C11	0.0375 (12)	0.0192 (10)	0.0499 (13)	−0.0019 (9)	0.0035 (10)	−0.0067 (9)
C12	0.0253 (9)	0.0280 (11)	0.0268 (10)	−0.0007 (8)	0.0105 (7)	−0.0003 (8)
C8'	0.0246 (12)	0.0373 (15)	0.0238 (12)	−0.0072 (11)	0.0004 (9)	−0.0080 (10)
N7'	0.0203 (12)	0.0291 (11)	0.0196 (10)	0.0044 (9)	0.0009 (8)	−0.0033 (8)
C10'	0.0343 (14)	0.0395 (15)	0.0208 (12)	0.0030 (12)	0.0012 (10)	0.0070 (11)
O4a	0.0291 (17)	0.0285 (10)	0.0324 (12)	0.0014 (12)	0.0137 (14)	0.0031 (9)
O5a	0.0384 (14)	0.024 (2)	0.0182 (8)	0.0011 (16)	0.0057 (8)	0.0039 (8)
N6a	0.0225 (9)	0.0340 (13)	0.0196 (8)	−0.0005 (9)	0.0058 (7)	0.0031 (8)
O7a	0.0333 (12)	0.107 (4)	0.0236 (9)	0.0220 (19)	0.0004 (8)	0.0030 (16)
O4b	0.0258 (18)	0.0284 (13)	0.0355 (12)	−0.0011 (11)	0.0127 (13)	−0.0027 (9)
O5b	0.0351 (17)	0.025 (2)	0.0221 (9)	−0.0064 (13)	0.0092 (9)	0.0025 (9)
N6b	0.0223 (10)	0.0307 (12)	0.0237 (9)	0.0025 (8)	0.0083 (7)	0.0051 (7)
O7b	0.0629 (19)	0.070 (3)	0.0359 (14)	0.0389 (19)	0.0218 (12)	0.0258 (17)

### Geometric parameters (Å, º)

**Table:**

Cd1—N1	2.3456 (16)	C5—N7'	1.483 (6)
Cd1—N2	2.5084 (14)	C5—C10'	1.517 (5)
Cd1—N3	2.3430 (19)	C5—H1c5	0.96
Cd1—N4	2.3598 (17)	C5—H2c5	0.96
Cd1—N5	2.3970 (17)	C5—H1c5'	0.96
Cd1—N7	2.426 (2)	C5—H2c5'	0.96
Cl1—O1	1.4276 (17)	C6—H1c6	0.96
Cl1—O2	1.426 (2)	C6—H2c6	0.96
Cl1—O3	1.4341 (15)	C7—C10	1.494 (4)
Cl1—O6	1.423 (2)	C7—C10'	1.517 (4)
N1—C2	1.481 (2)	C7—H1c7	0.96
N1—H1n1	0.870 (17)	C7—H2c7	0.96
N1—H2n1	0.870 (12)	C7—H1c7'	0.96
N2—C1	1.477 (3)	C7—H2c7'	0.96
N2—C6	1.483 (2)	C8—C9	1.534 (4)
N2—H1n2	0.870 (17)	C8—H1c8	0.96
N3—C7	1.476 (3)	C8—H2c8	0.96
N3—H1n3	0.870 (19)	C9—C11	1.510 (3)
N3—H2n3	0.870 (9)	C9—C8'	1.516 (7)
N4—C12	1.481 (3)	C9—H1c9	0.96
N4—H1n4	0.870 (5)	C9—H2c9	0.96
N4—H2n4	0.870 (15)	C9—H1c9'	0.96
N5—C11	1.485 (3)	C9—H2c9'	0.96
N5—H1n5	0.870 (17)	C10—H1c10	0.96
N5—H2n5	0.870 (14)	C10—H2c10	0.96
C1—C4	1.527 (3)	C11—H1c11	0.96
C1—H1c1	0.96	C11—H2c11	0.96
C1—H2c1	0.96	C12—H1c12	0.96
N7—C5	1.474 (3)	C12—H2c12	0.96
N7—C8	1.476 (4)	C8'—N7'	1.482 (9)
N7—H1n7	0.87	C8'—H1c8'	0.96
C2—C3	1.517 (3)	C8'—H2c8'	0.96
C2—H1c2	0.96	N7'—H1n7'	0.87
C2—H2c2	0.96	C10'—H1c10'	0.96
C3—C6	1.520 (3)	C10'—H2c10'	0.96
C3—H1c3	0.96	O4a—N6a	1.259 (6)
C3—H2c3	0.96	O5a—N6a	1.259 (7)
C4—C12	1.521 (3)	N6a—O7a	1.233 (8)
C4—H1c4	0.96	O4b—N6b	1.259 (15)
C4—H2c4	0.96	O5b—N6b	1.259 (13)
C5—C10	1.536 (4)	N6b—O7b	1.233 (15)
			
N1—Cd1—N2	81.40 (5)	C10'—C5—H1c5'	109.4712
N1—Cd1—N3	97.40 (6)	C10'—C5—H2c5'	109.4707
N1—Cd1—N4	159.83 (5)	H1c5—C5—H2c5	104.1342
N1—Cd1—N5	85.80 (6)	H1c5'—C5—H2c5'	108.7286
N1—Cd1—N7	94.22 (7)	N2—C6—C3	114.58 (16)
N2—Cd1—N3	89.96 (5)	N2—C6—H1c6	109.4713
N2—Cd1—N4	81.42 (5)	N2—C6—H2c6	109.4717
N2—Cd1—N5	99.37 (5)	C3—C6—H1c6	109.4713
N2—Cd1—N7	175.31 (7)	C3—C6—H2c6	109.4711
N3—Cd1—N4	93.06 (6)	H1c6—C6—H2c6	103.8245
N3—Cd1—N5	170.51 (5)	N3—C7—C10	114.4 (2)
N3—Cd1—N7	88.95 (7)	N3—C7—C10'	117.7 (4)
N4—Cd1—N5	86.66 (6)	N3—C7—H1c7	109.4717
N4—Cd1—N7	103.19 (7)	N3—C7—H2c7	109.4714
N5—Cd1—N7	81.89 (7)	N3—C7—H1c7'	109.4709
O1—Cl1—O2	110.06 (11)	N3—C7—H2c7'	109.4713
O1—Cl1—O3	108.51 (10)	C10—C7—H1c7	109.4709
O1—Cl1—O6	109.52 (12)	C10—C7—H2c7	109.471
O2—Cl1—O3	109.33 (10)	C10'—C7—H1c7'	109.4708
O2—Cl1—O6	109.28 (13)	C10'—C7—H2c7'	109.4719
O3—Cl1—O6	110.13 (12)	H1c7—C7—H2c7	104.0001
Cd1—N1—C2	120.21 (12)	H1c7'—C7—H2c7'	99.7563
Cd1—N1—H1n1	103.1 (15)	N7—C8—C9	115.1 (2)
Cd1—N1—H2n1	109.6 (15)	N7—C8—H1c8	109.4705
C2—N1—H1n1	110.9 (14)	N7—C8—H2c8	109.4704
C2—N1—H2n1	108.0 (15)	C9—C8—H1c8	109.472
H1n1—N1—H2n1	103.9 (19)	C9—C8—H2c8	109.4716
C1—N2—C6	109.29 (15)	H1c8—C8—H2c8	103.1391
C1—N2—H1n2	108.8 (14)	C8—C9—C11	116.97 (19)
C6—N2—H1n2	103.9 (18)	C8—C9—H1c9	109.4708
Cd1—N3—C7	120.15 (13)	C8—C9—H2c9	109.4714
Cd1—N3—H1n3	107.3 (13)	C11—C9—C8'	109.8 (4)
Cd1—N3—H2n3	109.0 (18)	C11—C9—H1c9	109.4713
C7—N3—H1n3	105.4 (13)	C11—C9—H2c9	109.4715
C7—N3—H2n3	108.6 (18)	C11—C9—H1c9'	109.4714
H1n3—N3—H2n3	105 (2)	C11—C9—H2c9'	109.4713
Cd1—N4—C12	108.81 (11)	C8'—C9—H1c9'	109.4716
Cd1—N4—H1n4	112.8 (18)	C8'—C9—H2c9'	109.4711
Cd1—N4—H2n4	108.3 (14)	H1c9—C9—H2c9	100.7569
C12—N4—H1n4	110.1 (17)	H1c9'—C9—H2c9'	109.1632
C12—N4—H2n4	107.0 (15)	C5—C10—C7	114.6 (2)
H1n4—N4—H2n4	110 (2)	C5—C10—H1c10	109.4713
C11—N5—H1n5	108.0 (15)	C5—C10—H2c10	109.4714
C11—N5—H2n5	107.8 (16)	C7—C10—H1c10	109.4714
H1n5—N5—H2n5	112 (2)	C7—C10—H2c10	109.4708
N2—C1—C4	114.00 (17)	H1c10—C10—H2c10	103.7599
N2—C1—H1c1	109.4715	N5—C11—C9	111.58 (18)
N2—C1—H2c1	109.4717	N5—C11—H1c11	109.4716
C4—C1—H1c1	109.4711	N5—C11—H2c11	109.4713
C4—C1—H2c1	109.4708	C9—C11—H1c11	109.4709
H1c1—C1—H2c1	104.5246	C9—C11—H2c11	109.4714
C5—N7—C8	109.52 (19)	H1c11—C11—H2c11	107.2787
C5—N7—H1n7	105.7778	N4—C12—C4	112.45 (17)
C8—N7—H1n7	111.2224	N4—C12—H1c12	109.4711
N1—C2—C3	112.75 (17)	N4—C12—H2c12	109.4712
N1—C2—H1c2	109.4715	C4—C12—H1c12	109.4712
N1—C2—H2c2	109.4715	C4—C12—H2c12	109.4713
C3—C2—H1c2	109.4715	H1c12—C12—H2c12	106.3151
C3—C2—H2c2	109.471	C9—C8'—N7'	115.9 (5)
H1c2—C2—H2c2	105.9748	C9—C8'—H1c8'	109.4709
C2—C3—C6	116.02 (16)	C9—C8'—H2c8'	109.4711
C2—C3—H1c3	109.4715	N7'—C8'—H1c8'	109.4716
C2—C3—H2c3	109.4709	N7'—C8'—H2c8'	109.4716
C6—C3—H1c3	109.4716	H1c8'—C8'—H2c8'	102.2362
C6—C3—H2c3	109.4707	C5—N7'—C8'	106.6 (4)
H1c3—C3—H2c3	102.0223	C5—N7'—H1n7'	111.4805
C1—C4—C12	115.64 (18)	C8'—N7'—H1n7'	108.0034
C1—C4—H1c4	109.4709	C5—C10'—C7	114.4 (2)
C1—C4—H2c4	109.4712	C5—C10'—H1c10'	109.4716
C12—C4—H1c4	109.4715	C5—C10'—H2c10'	109.4715
C12—C4—H2c4	109.4715	C7—C10'—H1c10'	109.4715
H1c4—C4—H2c4	102.5005	C7—C10'—H2c10'	109.4708
N7—C5—C10	114.33 (19)	H1c10'—C10'—H2c10'	104.0234
N7—C5—H1c5	109.471	O4a—N6a—O5a	119.5 (5)
N7—C5—H2c5	109.4715	O4a—N6a—O7a	120.2 (5)
C10—C5—H1c5	109.4711	O5a—N6a—O7a	120.3 (6)
C10—C5—H2c5	109.4712	O4b—N6b—O5b	119.5 (10)
N7'—C5—C10'	110.2 (5)	O4b—N6b—O7b	120.2 (10)
N7'—C5—H1c5'	109.4712	O5b—N6b—O7b	120.3 (12)
N7'—C5—H2c5'	109.4718		

### Hydrogen-bond geometry (Å, º)

**Table:**

*D*—H···*A*	*D*—H	H···*A*	*D*···*A*	*D*—H···*A*
N1—H1*n*1···O5*a*^i^	0.870 (17)	2.289 (19)	3.127 (6)	161.7 (16)
N1—H1*n*1···O5*b*^i^	0.870 (17)	2.10 (2)	2.929 (10)	158.1 (16)
N1—H2*n*1···O7*a*^ii^	0.870 (12)	2.278 (17)	2.986 (7)	139 (2)
N1—H2*n*1···O4*b*^ii^	0.870 (12)	2.403 (14)	3.262 (9)	170 (2)
N1—H2*n*1···O7*b*^ii^	0.870 (12)	2.410 (19)	3.027 (11)	128.4 (19)
N2—H1*n*2···O4*a*^i^	0.870 (17)	2.339 (18)	3.149 (4)	155.0 (18)
N2—H1*n*2···O4*b*^i^	0.870 (17)	2.28 (2)	3.116 (10)	162.4 (16)
N3—H1*n*3···O3^iii^	0.870 (19)	2.43 (2)	3.196 (3)	148 (2)
N3—H2*n*3···O5*a*^iv^	0.870 (9)	2.472 (13)	3.289 (8)	157 (2)
N3—H2*n*3···O7*a*^iv^	0.870 (9)	2.48 (2)	3.134 (7)	132 (2)
N3—H2*n*3···O5*b*^iv^	0.870 (9)	2.426 (18)	3.215 (13)	151 (2)
N3—H2*n*3···O7*b*^iv^	0.870 (9)	2.50 (2)	3.239 (13)	144 (2)
N5—H1*n*5···O4*a*^i^	0.870 (17)	2.396 (16)	3.200 (4)	154 (2)
N5—H1*n*5···O5*a*^i^	0.870 (17)	2.30 (2)	3.065 (7)	146.5 (16)
N5—H1*n*5···O5*b*^i^	0.870 (17)	2.33 (2)	3.076 (12)	144.0 (16)
N5—H2*n*5···O1^v^	0.870 (14)	2.263 (14)	3.122 (2)	169 (2)
N7—H1*n*7···O4*a*^ii^	0.87	2.20	3.067 (4)	174.11
N7—H1*n*7···O4*b*^ii^	0.87	2.45	3.313 (11)	169.55
N4—H1*n*4···O3^iii^	0.870 (5)	2.379 (11)	3.215 (2)	161 (2)
N4—H2*n*4···O2^v^	0.870 (15)	2.170 (15)	3.011 (2)	163 (2)
C10—H1*c*10···O7*b*^ii^	0.96	2.46	3.407 (13)	170.52
C8′—H2*c*8′···O4*a*^ii^	0.96	2.12	3.069 (11)	168.87
C8′—H2*c*8′···O4*b*^ii^	0.96	2.42	3.364 (15)	168.03
C7—H1*c*7···O7*a*^iv^	0.96	2.48	3.067 (7)	119.01
C7—H1*c*7′···O7*a*^iv^	0.96	2.44	3.067 (7)	122.52

Symmetry codes: (i) −*x*+1, −*y*+1, −*z*+1; (ii) −*x*+1, *y*+1/2, −*z*+1/2; (iii) *x*, *y*+1, *z*; (iv) *x*, −*y*+3/2, *z*−1/2; (v) −*x*, *y*+1/2, −*z*+1/2.
